# Back from the rabbit hole. Theoretical considerations and practical guidelines on psychedelic integration for mental health specialists

**DOI:** 10.3389/fpsyg.2023.1054692

**Published:** 2023-10-12

**Authors:** Jakub Greń, Filip Tylš, Michał Lasocik, Csaba Kiraly

**Affiliations:** ^1^Public Health Department, Institute of Psychiatry and Neurology, Warsaw, Poland; ^2^Polish Psychedelic Society, Warsaw, Poland; ^3^Psyon - Psychedelic Clinic, Prague, Czechia; ^4^3rd Medical Faculty, Charles University in Prague, Prague, Czechia; ^5^National Institute of Mental Health, Klecany, Czechia; ^6^Czech Psychedelic Society, Prague, Czechia; ^7^Polish Society of Process Psychology, Warsaw, Poland; ^8^Multidiszciplináris Társaság a Pszichedelikumok Kutatásáért, Budapest, Hungary

**Keywords:** psychedelics, psychedelic integration, psychedelic-assisted therapy, clinical practice, guidelines

## Abstract

The growing interest in and prevalence of the use of psychedelics, as well as the potential benefits and negative consequences associated with psychedelic experiences, create a need for mental health specialists to be able to provide adequate and effective intervention regarding the content and consequences of these experiences, that is, psychedelic integration. At the same time, current graduate training in psychiatry, psychology, psychotherapy, counseling, etc., fails to adequately prepare professionals for such interventions. In order to fill this gap, an international, bottom-up project was established to attempt developing guidelines. This project was conducted by means of literature reviews as well as roundtable discussions among project participants, leading to a consensus on the guidelines’ final scope and content. Drawing from the outcomes of this project, this article presents proposed comprehensive guidelines covering both theoretical and practical aspects of psychedelic integration, that are intended to serve as a resource for various mental health specialists who may encounter individuals in need of support considering their psychedelic experiences. These guidelines encompass clinician-friendly information on the effects of psychedelics, a definition of psychedelic integration, the general theoretical considerations linked to utilization of psychedelic experiences in clinical practice, a simple model organizing the course of psychedelic integration practice, as well as an overview of the current models of psychedelic integration, along with a selective presentation of basic and specific interventions derived from various psychotherapeutic approaches that can be employed in the practice of psychedelic integration.

## Background

### Mind manifestations

*“Psychedelics”* is an umbrella term that refers to a heterogeneous group of psychoactive substances^1^ known for inducing a broad range of specific effects on various aspects of human experiences (for review see: Nichols, 2016; Swanson, 2018). These effects include perceptual (primarily visual) changes with both open and closed eyes (e.g., distortions, mental imagery); intensified emotional reactivity along with the increased access to one’s emotions; paradoxical cognitive changes, with acute cognitive impairments on one side and increased creativity on the other; as well as alteration in one’s sense of self, body, surroundings and time (e.g., the experience of ego-dissolution, and the loss of a sense of boundaries between self and world; Millière, 2017). These various effects make up specific altered states of consciousness called *psychedelic experiences,* the etymological meaning of which might be translated as “mind-manifesting experiences”,^2^ as it refers to the phenomenon of pulling the unconscious material to the conscious surface, allowing it to be accessed and processed (Osmond, 1957).

### Therapeutic potentials of psychedelics

The acute effects of psychedelics are often accompanied by insights and the attribution of high personal meaning and extraordinary significance (Griffiths et al., 2006), which indicates the therapeutic, and perhaps transdiagnostic, potentials of these substances (Garcia-Romeu and Richards, 2018; Aday et al., 2020). A growing body of evidence suggests that outcomes of psychedelic experiences might yield long-term and mostly beneficial results on mental health problems (i.e., alleviating depression or anxiety symptoms, breaking patterns of addictive behaviors), even following a single dose (Andersen et al., 2021). Other studies reported a positive impact of these experiences on personality traits, as well as more general psychological functioning and well-being (i.e., Griffiths et al., 2006; Bouso et al., 2015; Hendricks et al., 2015), and have even shown potential for reducing recidivism among individuals in the criminal justice system (Hendricks et al., 2014). It should be noted, however, that in the absence of supportive and sustaining efforts, the potential benefits of psychedelic use tend to fade over time (Aday et al., 2020). On the other hand, in spite of relatively high levels of physiological safety, psychedelic experiences can be very challenging and may lead to numerous adverse consequences that are both short- or long-term (Carbonaro et al., 2016).

### Adverse psychedelic-related consequences

The intense, confusing, and often overwhelming nature of psychedelic effects commonly results in challenging experiences, which can lead to various adverse outcomes (Carbonaro et al., 2016; Lutkajtis and Evans, 2023). These outcomes include acute-stress- or trauma-like symptoms, such as distress, disturbing thoughts, and feelings, mood fluctuations, dissociative experience of derealization or depersonalization, as well as an intrusive re-living of the challenging experience as occasional “flashbacks” or nightmares. The emergence of traumatic memory (e.g., a memory of both known or previously unknown very difficult events/experiences that could not be coped with in the moment) can also occur during the psychedelic experience (Aixalà, 2022).

In this context, some authors refer also to the “ontological shock,” which is a state of being forced to question one’s worldview, which might result from the radical alternation of everyday perception and informational overload often occurring under the influence of psychedelics (Carhart-Harris and Friston, 2019).

Further, the concept of “spiritual bypassing,” though not psychedelic-specific, is increasingly invoked in the context of psychedelics as one of the indicators of non-integrated psychedelic experiences (Gorman et al., 2021; Aixalà, 2022; Lutkajtis and Evans, 2023). Spiritual bypassing refers to a tendency to avoid addressing unresolved problems, dealing with “mundane matters” or neglecting close relationships and responsibilities, due to declared occupation with spiritual or transpersonal ideas and development.

If persistent and not properly cared for, adverse psychedelic-related consequences can cause chronic distress, anxiety, and impairment in everyday life (Phelps, 2017; Lutkajtis and Evans, 2023), as well as suicide attempts among vulnerable individuals (Schlag et al., 2022). Here we should also mention a hallucinogen persisting perception disorder (HPPD), a psychedelic-specific condition characterized by prolonged or recurrent sensory impairments and hallucination-like symptoms (Halpern et al., 2016). Although the occurrence of such adverse outcomes is relatively uncommon, they constitute a range of potential risks associated with the use of psychedelics.

In sum, several adverse psychedelic-related consequences are being reported among people using psychedelics, which speak to the nature of psychedelic experiences that can induce a vulnerable state both during and after the acute effects (Andersen et al., 2021).

### Psychedelic research and therapy

The investigation of psychedelic experiences and their therapeutic application dates back three decades prior to their scheduling as prohibited in the early 1970s (Rucker et al., 2018). After a legally imposed hiatus in research and clinical application, scientific investigation (known as the *psychedelic renaissance*; Sessa, 2012) has resumed, and these substances are now increasingly recognized by the academic, medical, and psychotherapeutic communities as substances with high potential in the treatment of many mental health conditions (Garcia-Romeu and Richards, 2018; Inserra et al., 2021). Applied research on psychedelics, however, is not merely a present-day phenomenon. Between 1940 and 1970, these substances were the subject of numerous studies focused on their clinical use as an adjunct to pharmacological treatments of alcohol use disorder, psychosis, and neurosis (for review see: Rucker et al., 2018).

Psychoanalytic theory, which dominated clinical practice at that time, contributed to the development of *psycholytic therapy* in the 1960s. This approach to working with psychedelics involved the repeated use of low-to-medium doses of psychedelics in the clinical context to facilitate psychoanalytic therapy (for review see: Passie et al., 2022). In parallel, another approach – *psychedelic-assisted therapy* (PAT) – has also been developed, which more explicitly combines psychiatric and psychotherapeutic interventions. Specifically, PAT consists of the administration of psychedelics in medium-to-large doses to provoke intense psychedelic experiences under controlled clinical settings. This is preceded by screening and psychological preparation, as well as followed by so-called psychedelic integration (see “What is psychedelic integration?” in the Theoretical considerations section).

### The need for psychedelic integration

Given the aforementioned potentially beneficial effects as well as risks and challenges associated with psychedelic experiences, there is a growing need for psychedelic integration practice. This practice might be defined as clinical/psychotherapeutic practice focused specifically on the content and/or consequences of psychedelic experiences. The need for psychedelic integration also stems from media and market interest (or even “hype”) related to promising early results of medical application of psychedelics, which has its reflection in the growing interest in and prevalence of psychedelic substances use in various settings, which might be loosely divided into legal and illegal (Rucker et al., 2018; Sexton et al., 2019; Yaden et al., 2022a; Noorani et al., 2023). The first one includes patients of the growing market of ketamine clinics and participants in clinical trials focused on PAT. In the latter case, Noorani et al. (2023), have recently pointed to the emergence of local community-based support groups with regard to psychedelic integration, among participants of PAT-focused clinical trials. Whereas the second refers to participants of underground therapies, psychedelic retreats, people who self-experiment with psychedelics (i.e., psychonauts), or those who use psychedelics in recreational settings (e.g., outdoor music festivals). This is illustrated for instance in the study by Killion et al. (2021) who analyzed data from nationally-representative cross-sectional studies among a total of 664,152 adult United States civilians, and found that LSD use has increased by 200% between 2002 and 2018.

At the same time, there is a lack of formal preparation for mental health professionals (i.e., psychiatrists, clinical psychologists, psychotherapists, counselors, etc.) who will have increasing contact with clients having psychedelic experiences (e.g., Phelps, 2017). Thus, the first proposals for psychedelic integration models have already emerged, though they are focused on specific contexts of psychedelic use, clinical trials, and ceremonial use in particular (for review see: Bathje et al., 2022). Several training centers have recently started offering workshops or certification courses on psychedelic integration or PAT, and work is currently underway to establish multi-university postdoctoral fellowships and training programs in psychedelic therapy.^3^ However, to date, these are all postgraduate training courses and primarily in the US, which is unlikely to meet the growing demand. Moreover, with the exception of Gorman et al. (2021), who published their proposed model of psychedelic integration practice as a foundation for training in this regard,^4^ none of the training centers/companies developed (or made public) psychedelic integration guidelines.

### Purpose of this paper

Therefore, the purpose of this paper was to address the risks and needs associated with psychedelic experiences and outline our attempt to develop comprehensive guidelines on both theoretical and practical aspects of psychedelic integration. Such guidelines may inform the individual practice of various mental health specialists, who may encounter and work with clients in need of support with regard to the content and/or consequences of their psychedelic experiences.

## Guidelines development

Guidelines presented in this paper arose from the larger project entitled “An international network of therapists for integration of psychedelic experiences,” which was aimed at the development of the guidelines and training curriculum on psychedelic integration for the general mental health specialist audience (i.e., psychotherapists, psychologists, psychiatrists). This project was funded by the Visegrad Fund (Project ID: 21820342) and brought together an international team composed of mental health specialists and researchers who agreed to represent official institutions and societies related to psychedelics from the Visegrad group countries, including the Czech Republic, Poland, Hungary, and Slovakia, with support of partner organization from Germany. Specifically, our team consisted of psychotherapists representing various psychotherapeutic approaches, including humanistic (i.e., person-centered approach, Gestalt), psychodynamic and depth psychology (i.e., Jungian analytical approach and process-oriented psychology), a clinical psychiatrist and neuroscientist (Ph.D.), as well as an addiction therapist and harm reductionist. Specifically, this team was composed of the authors of this paper and individuals listed in the Acknowledgements section. Our current affiliations included national psychiatric hospitals, private clinics, psychotherapeutic centers, clinically-oriented research centers, non-governmental organizations providing harm reduction services as well as national-level psychotherapeutic and psychedelic societies.

### Roundtable discussions

The initial guidelines were developed through the roundtable discussions and brain-storm exercises performed at the very beginning of our work. First roundtable discussion (in-person) aimed to define what “psychedelic,” “psychedelic experience,” “integration” and “psychedelic integration” means to each of us and what associations come up when we think of one of those terms. We then merged associations from each term and eventually came up with a consensual definition of psychedelic integration. In addition, we divided psychedelic integration practice in terms of its timeline as well as the scope of time after the psychedelic experience that integration practice takes place (i.e., early and late stages), as well as the theoretical and practical aspects of integration. Subsequently, after this first roundtable discussion, we split ourselves into four sub-groups (drawn on the experience in a particular theme) that were to explore and prepare a draft on four emergent areas: early theoretical, early practical, late theoretical, and late practical. These drafts were then compiled into one document which was the subject of discussion within the second roundtable meeting (in person) until the whole team had reached a consensus on the scope and content of the guidelines document.

### Guidelines document

After compilation, this document was informed by the findings of a literature review that was conducted by one of the authors of this manuscript. This led to a substantial refinement of the guidelines document, which consisted of:

- historical background of psychedelics, their use by humans, and psychedelic-focused research throughout the 20-century and at the beginning of 21-century (i.e., the psychedelic renaissance); psychedelic substances’ effects and mechanisms of action;
- overview of psychedelics’ therapeutic potentials and related adverse consequences;
- the rationale behind the need for psychedelic integration practice, guidelines, and training;
- the definition of psychedelic integration;
- our model of the course of psychedelic integration practice;
- theoretical basis and approaches of psychedelic integration;
- an overview of currently available models of psychedelic integration;
- description of the practical application of selected psychotherapeutic approaches and methods that we considered to be adequate and potentially useful for psychedelic integration practice.

Further discussions were then conducted over the guidelines document (this time online due to the restrictions related to the COVID-19 pandemic), the results of which formed a final guidelines document that we delivered to the project Funder, together with a formal report of project completion. This final guidelines document served as a basis for preparing the present article.

### Literature review

Literature review for the purpose of the guidelines document was conducted using PubMed, Web of Science, and Scopus research databases, as well as Google Scholar, through September 1, 2020. The keywords were: “psychedelic integration” and “psychedelic therapy.” At first, the attempt to conduct a systematic literature review restricted to the available peer-reviewed literature was taken, but it resulted in identifying only one article that specifically considered psychedelic integration (Watts and Luoma, 2020). Thus, a comprehensive literature review was adopted that also included clinical trial manuals and books. Preprints, dissertations, magazine articles, and other gray literature were not included, though we report that numerous examples of such literature concerning psychedelic integration and therapy exist. The same comprehensive literature review was re-conducted after the project completion when the initial manuscript was being prepared based on the previously developed guidelines document. This second literature review was completed by August 25, 2022, and it resulted in inclusion in the manuscript of several recently published literature positions on the subject of psychedelic integration.

## Theoretical considerations

### What is psychedelic integration?

The term “integration” is used in various contexts and it usually refers to “connecting” or “bringing together” something that is or has become separated. By the “integration of psychedelic experience” in the clinical or therapeutic setting we understand any conscious and informed attempt to facilitate processing the psychedelic experience content or resulting consequences, in order to implement its relevant outcomes into everyday life. This relevant content might include a particular insight, be it cognitive or emotional (i.e., memory, vision), as well as the very fact that whatever was experienced under the influence of psychedelics, its subjective realness and meaningfulness might not be regarded as something to downplay or ignore.

Our definition of psychedelic integration coincides with most other definitions that can be found in current literature. Specifically, Earleywine et al. (2022) used a mixed-method approach to determine common themes of psychedelic integration definitions provided by various therapists offering psychedelic integration in different approaches. The sample consisted of 30 therapists (50% male), who lived in American countries (70% in the United States), identified mostly as White (73,3%), and were aged between 29 and 69 (*M* = 40.66; SD = 12.08). The authors found that participants viewed integration as a bridge between the psychedelic experience and daily life, which is a process that ideally begins prior to the substance intake, never ends, makes sense of the psychoactive experience, creates behavioral change, is personalized, and makes the individual whole. In turn, Bathje et al. (2022) proposed the following synthesized definition of psychedelic integration based on their extensive literature review:

“a process in which a person revisits and actively engages in making sense of, working through, translating, and processing the content of their psychedelic experience. Through intentional effort and supportive practices, this process allows one to gradually capture and incorporate the emergent lessons and insights into their lives, thus moving toward greater balance and wholeness, both internally (mind, body, and spirit) and externally (lifestyle, social relations, and the natural world)”.

### Is it solely (in) the substance?

**Figure 1:**
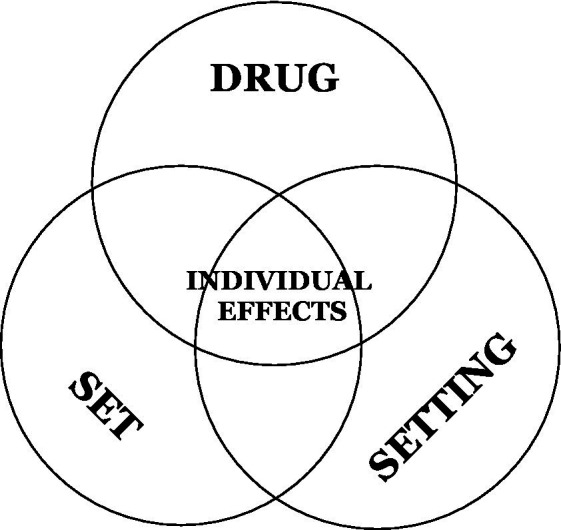
The “drug, set and setting” model of individual effects of psychoactive substances.

The intensity, duration, outcomes, and subjective attribution of experiences induced by psychedelics depend not only on the particular substance type or its dose but also largely on extra-pharmacological factors. These factors include *set* (individual traits, pre-state, and expectations toward substance use/effects) and *setting* (the physical and social environment in which the substance is taken). The “drug, set and setting” model (depicted in Figure 1) of individual effects of psychoactive substances (Zinberg, 1984) may be particularly relevant in the case of psychedelics (Carhart-Harris et al., 2018). In fact, the term “non-specific amplifiers” has been used to describe the dynamic process and largely unpredictable outcome of these substances, emphasizing the action of psychedelics by amplification of set and setting, rather than producing hallucinations (Grof, 1976).

The influence the side of the substance, set, and setting should not be underestimated as they clearly play a role in both the quality and potential risk of psychedelics use. In fact, Olson et al. (2020) reported considerable individual variation in the psychedelic-like effects of placebo, which were promoted by expectations (set) and context (setting). Further, according to existing research, factors like high personality trait of neuroticism, personal/family history of psychosis, borderline personality, mania or related psychiatric conditions, inappropriate context, being an unexperienced user of a particular substance, as well as lack of or insufficient post-experience integration, are the most commonly associated with adverse reactions (Johnson et al., 2008; Barrett et al., 2017; Ona, 2018).

The role of these factors should not be limited to preparation for or being under the influence of psychedelics but needs to be taken into account during the integration process as well (i.e., as a part of discussing and interpreting the course and content of client’s psychedelic experience), which is elaborated in the practical guidelines section.

### Should therapists have their own psychedelic experiences?

The issue of whether one’s first-hand experience with the effects of psychedelics among those working with psychedelic experiences (i.e., therapist) is necessary or not is the subject of ongoing debate in the field (Nielson and Guss, 2018). We believe that first-hand experiences with psychedelic substances are an important consideration also among mental health specialists offering psychedelic integration and not just PAT. Such experiences can provide personal insight into the nature of psychedelic effects, which contribute to understanding clients’ experiences and related difficulties in the way that reading guidelines such as these, or undergoing clinical training alone, cannot provide. This view is based on other authors’ recommendations for psychedelic therapists to have their own experiences with non-ordinary states of consciousness, be it with psychedelic compounds or with a variety of non-pharmacological methods, such as Holotropic Breathwork (for review see: Phelps, 2017).

However, we do not assume that personal use of psychedelics is necessary to provide support with regard to the content and/or consequences of a client’s psychedelic experiences. It should also be emphasized that psychedelic self-experience is not always possible or advisable due to the illegal status of these substances in most countries worldwide, or due to potential contraindications for a given individual. Further, Nielson and Guss (2018) have pointed out that a therapist’s first-hand experience with psychedelics, while common in the history of medical research on psychedelics, is largely omitted, undocumented, and understudied, which indicates the need for reliable investigations of the prevalence and influence of this phenomenon.

### How do psychedelics work?

Classical psychedelics (i.e., psilocybin, mescaline, LSD, DMT) act by interaction (i.e., agonism) with serotonin receptors in the brain (Tylš et al., 2014), which causes a cascade of various biochemical, neural, and psychological mechanisms underlying psychedelic effects (for review see: Van Elk and Yaden, 2022). Regarding the effects and underlying mechanisms of non-classical psychedelics (i.e., MDMA or ketamine) we refer to the extensive review by Nichols (2016).

Under the influence of psychedelics, the disruption of normal brain connectivity and hierarchy is observed (Carhart-Harris et al., 2013), which includes the reduction of the inhibitory influence of evolutionarily more developed parts (such as the frontal cortex). Specifically, the frontal brain regions are separated from the temporal regions, which then activate evolutionary older “operators” that are responsible for the predominance of so-called *primary thinking* (Carhart-Harris et al., 2014; Swanson, 2018). This pattern of thinking is characterized by irrationality, ambiguity, conceptual paradox, and intense or impulsive emotions. Moreover, decreased frontal dominance over limbic areas precipitates the emergence of an unconscious content that is suppressed under normal conditions by *secondary thinking*, which in turn is characterized by order, precision, conceptual consistency, and controlled affect. Thus, primary thinking might reveal some important insights that are normally inhibited. However, after psychedelic effects expire, the activation of more rational and evolutionary younger secondary thinking is needed in processing, understanding, and integrating the experience properly.

Despite the substance disappearing from the body, changes triggered by psychedelics on the level of the brain network persist long-term (Carhart-Harris et al., 2014; Carhart-Harris and Friston, 2019). This concerns primarily neuroplasticity, which is the ability of brain neural networks to change through growth, forming new connections, and reorganization (Jones et al., 2009; Szabo et al., 2016; Dakic et al., 2017; Ly et al., 2018). At the psychological level, it is accompanied by increased sensibility, suggestibility, and empathy, in the weeks following the psychedelic experience, which further supports the learning process.

The psychological correlates of these changes are known as the *afterglow effect*. According to Majić et al. (2015), this may last about 6 to 8 weeks after the consumption of psychedelics and is usually characterized by slightly elevated mood, optimism, and higher openness, as well as weakened ego defense mechanisms resulting in higher vulnerability. Due to the increased neural and psychological flexibility during this period (Davis et al., 2020), new cognitive patterns or mental representations may be more likely to form, which is essential for the process of psychedelic integration. However, for the same reasons, the heightened suggestiveness of the client needs to be respected (i.e., any kind of dogmatic point of view or interventions that were not consented to need to be avoided during the client’s afterglow period).

### Trust the inner healer or be directive?

In the current literature on PAT, there is a distinction between non-directive and directive approaches (Hendy, 2022). The former regards the concept commonly known as *inner healer* or *inner healing intelligence*, which is often invoked in the context of psychedelic experiences as an attempt to explain their broad therapeutic effects. It is also a vital underpinning of approaches utilizing non-ordinary states of consciousness, like Holotropic Breathwork (Grof, 2014) or mindfulness-based therapy (Baer, 2015).

The inner healer concept is built on the assumption about the internal and involuntary tendency that guides the process of the psychedelic experience. This tendency is reflected in the fact that during a psychedelic experience, salient but difficult (and therefore previously suppressed) content can emerge to the surface of consciousness, allowing them to be processed (i.e., Carhart-Harris et al., 2014; Watts and Luoma, 2020). In this context, Gorman et al. (2021) have cited Rick Doblin’s description regarding the utilization of this inner-directed in the MDMA-assisted therapy clinical trials:

“We all know that’s true for our bodies. If you get a scratch or break bones, your body has a mechanism to heal itself… There is this wisdom of the body to try to sustain itself. We think similarly there is something like that for the psyche”.

Nonetheless, several authors have provided criticism of the reliance on this approach in research on the therapeutic application of psychedelics. This critique concerns limitations related to the theoretical foundation and the implications of the inner healer concept (i.e., bias toward concentration on intrasubjectivity), as well as the influence of transpersonal theory, perennial philosophy, and new-age culture, which lack strong empirical support (Roseman et al., 2021). Others also pointed out that relying on the notion of the inner healer leads researchers and clinicians to focus on the psychedelic experience itself while providing only nonspecific psychological support, and this neutrality has been questioned (Guss et al., 2020). Moreover, this might also prevent the utilization of coherent therapeutic approaches, the standardized and theorized methods of which could be tailored to a given individual.

Here we do not want to resolve the tension between these perspectives. Rather, we intend to inform the reader about this ongoing discussion and argue that, from our perspective, both non-directive and directive approaches can be useful in psychedelic integration practice (for one such example see “Resource orientation” in the Practical guidelines section). In our view, at the early stages of processing a psychedelic experience, the client should be provided with the unrestricted space to express its content both verbally and nonverbally (if only because of the often ineffable nature of these experiences). However, in order to facilitate the process of understanding the experience, extracting its insight, translating it into behavioral changes, and supporting their implementation in daily life, both more structure and directivity on the part of the therapist, as well as the use of approaches and methods consistent with a given school of psychotherapy, will likely be the most effective.

### Is psychedelic integration legal when psychedelics are not?

Due to the illegality of psychedelic substances in most countries worldwide, psychedelic integration practice is surrounded by a number of ethical considerations (for review and recommendations see: Pilecki et al., 2021). These are dependent on local drug policy and license specification, but among them, one can be listed: being the subject of disciplinary action taken by the licensing board to which the practitioner is affiliated (that may see it as unprofessional, unethical, or outside of the boundaries of acceptable practice), as well as potential accusation of malpractice by the client (who may feel somehow disadvantaged) or, for instance, his/her family members (who may not be in favor of psychedelic integration).

These considerations are similar to those that arise when working with individuals who continue to use other illicit psychoactive substances, as in the case of the harm reduction-based model of addiction psychotherapy (Tatarsky, 2003). *Harm reduction* refers to evidence-based, pragmatic, compassionate, and non-judgmental approaches to policy, prevention, or clinical interventions that are focused on reducing negative consequences without eliminating their source altogether. In the case of substance addiction, this means focusing on modifying the pattern of use to promote safety and reduce related harms, without necessarily stopping overall substance use if the client is unwilling or unable to abstain from it at that time for any reason. Thus, the harm reduction approach provides the theoretical basis and rationale for interventions and clinical practice regarding people who undertake problematic behaviors, such as illicit psychoactive substance use.

As proposed by Gorman et al. (2021) and Pilecki et al. (2021), the harm reduction approach may also be used as a framework to provide clinical practice around clients’ use of psychedelics, including psychedelic preparation and integration. Within this approach, the use of psychedelics is not pathologized or stigmatized, nor is it promoted or encouraged. Rather, a mental health specialist is focused on addressing a client’s needs, assessing and dealing with his/her psychedelic-specific and co-occurring issues (whether social, health, or legal), as well as on informing their decisions about future psychedelic use. For example, if a client is interested in using psychedelics in order to treat their mental health condition, a non-judgmental education about psychedelics’ effects, related risks and contraindications to their use should be provided. Moreover, following a harm reduction perspective, if there are more conventional methods of treating the client’s condition with proven effectiveness (such as pharmacotherapy and/or regular psychotherapy in a particular modality), they should be encouraged to use them instead of taking psychedelics, in order to avoid risks associated with their use. Likewise, a discussion should be had about engaging in alternative methods that utilize altered states of consciousness (e.g., Holotropic Breathwork, mindfulness-based therapy, hypnotherapy) that do not involve administration of the psychoactive substance (especially if it is illegal under local regulations).

### What about psychedelic preparation?

This paper is focused on psychedelic integration. However, ideally, a psychedelic experience is preceded by preparation for it. The value of psychedelic preparation should not be overlooked, as it is able to prevent some of the potential adverse consequences of using psychedelics. Also, according to Aixalà (2022), in cases where a lack of proper preparation (and/or experienced facilitation) resulted in adverse consequences, it might be helpful to provide an education that would serve as a sort of “retrospective preparation,” so that client would be aware of omissions or malpractices that he/she had experience. Guidelines on preparation for psychedelic experience have already been developed and presented in detail in numerous articles (e.g., Johnson et al., 2008; Carmo Carvalho et al., 2014; Gorman et al., 2021) or books (e.g., Fadiman, 2011; Westrum and Dufrechou, 2019), which is why this is not elaborated upon in this article. Here we only provide an outline of what such preparation should be or usually consists of (e.g., in the context of PAT or PAT clinical trials):

- *screening* for preexisting psychiatric disorders or other contraindicators that put individuals at high risk and therefore should discourage them from using psychedelics for safety reasons; this includes a current or past history of psychotic disorders, bipolar affective disorder, and/or borderline personality disorder, current medication (e.g., with antipsychotic medications, tricyclic antidepressants, lithium, serotonin reuptake inhibitors, monoamine oxidase inhibitors) or psychoactive substance use, pregnancy, serious neurological, renal, liver or cardiac disease, as well as such relative contraindicators as family history of psychosis or suicide attempts (for review see: Johnson et al., 2008)
- *psychoeducation* (i.e., informing about the nature of psychedelic experience, and about how these substances affect the human brain and mind, as well as addressing the client’s questions and concerns);
- *counseling* (discussing the expectancies toward the psychedelic experience and supporting the emergence of personal intention for it);
- discuss *harm reduction measures* that might reduce the potential risks/harms in terms of set and setting during the experience (i.e., Fadiman, 2011).

## Practical guidelines

### The course of the psychedelic integration practice

**Figure 2:**
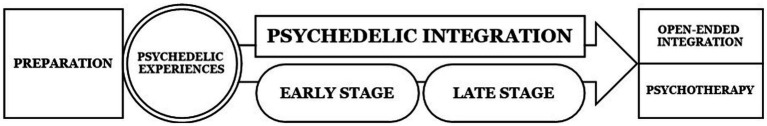
The simple two-stage model of the course of psychedelic integration practice.

As a way to organize psychedelic integration practice, we propose a simple two-stage model, as depicted in Figure 2. This model is conceptualized on the dimension of time, dividing psychedelic integration practice into early and later stages, which follow the psychedelic experience. This model also distinguishes psychedelic integration from regular psychotherapy (for more information see “After the integration” in the Practical guidelines section). We believe that this distinction should be made, as clients who might seek support with regard to their psychedelic experiences do not necessarily need or want to begin psychotherapy. In some cases, however, psychedelic integration might transition to psychotherapy, that is, to clinical practice that is not solely focused on the content and/or consequences of the client’s psychedelic experience(s). In other cases, psychedelic integration ends with what we like to call, an open-ended integration, which is meant to acknowledge both the possibility of renewed contact with the client later on (i.e., follow-up sessions) as well as the client’s own ongoing and autonomous work to integrate the psychedelic experience into his/her everyday life, which continue after such collaboration.

The *early stage* is assumed to be short-term (lasting approximately up to five one-hour sessions) and is intended to cover standard clinical/therapeutic practices. This includes providing basic support, establishing the therapeutic relationship, screening for conditions requiring medical management, psychoeducation, and normalization (if needed), as well as specifying the client’s goals and determining working direction. Treating the potential adverse psychedelic-related consequences and stabilizing the client’s condition should be prioritized here as it is usually the reason to seek such help. In fact, although psychedelic integration might be focused on maximizing and sustaining benefits arising from the psychedelic experience (see below), such work is usually performed within the context of psychedelic clinical trials, retreats, or organized ceremonies. Whereas people seeking help from therapists, or other mental health specialists offering clinical practice, usually do so due to adverse psychedelic-related consequences that they could not resolve by themselves (or with the help of previous healthcare providers). Thus, the “Basic interventions” described in practical guidelines are most suitable in the early stage, while “Specific intervention” may be more useful in the *late stage*, as it concerns how different psychotherapeutic approaches and methods might be adopted for the purpose of meeting client’s needs regarding psychedelic integration. While the early stage is assumed for rather quick and basic interventions (i.e., coping with confusion; providing psychological support), the late stage is focused on deeper work at a longer period (i.e., wider exploration of the specific content of the psychedelic experience; support in incorporating and maintaining major insights into one’s everyday life). In that sense, the late stage is a natural continuation of the previous work and could also be the beginning of regular psychotherapy, which may reach far beyond the scope of the client’s psychedelic experience.

### Integration goals

**Figure 3:**
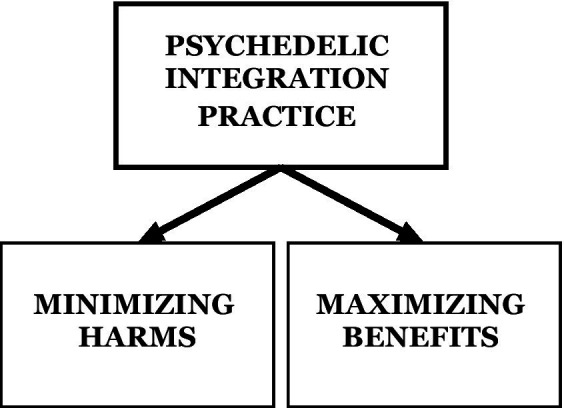
The general organization of the needs and/or goals of psychedelic integration practice.

For individuals seeking mental health specialists’ support regarding their psychedelic experience, the interventions should focus on its integration. From the pragmatic stance, the needs and/or goals of such interventions might be generally organized as either maximizing the benefits or minimizing the harms that result from the psychedelic experience in question (Figure 3). This distinction is commonly found in the literature on psychedelic integration (e.g., Gorman et al., 2021; Aixalà, 2022). However, each of the undertaken interventions should directly address the client’s articulated needs and concerns.

In the first case, we are dealing with an experience that is most probably perceived as positive and therefore beneficial for the client’s condition. The client may, for example, feel a notable improvement in his/her mood or may have broken an unfavorable pattern of behavior that has accompanied him/her for a long time. However, as the changes initiated by such an experience tend to fade over time, the client might need both conditions and specific practices that will enable them to consolidate and preserve his/her life. As a result of a psychedelic experience, a client might also experience undesirable symptoms that interfere with his/her condition and/or well-being. These symptoms might mean both a worsening of existing mental health problems, and experiencing some previously non-existent difficulties (e.g., anxiety, sleep disturbances, emotional reactivity, or impaired cognition). Then, integration work should be primarily focused on psychological support in relieving these symptoms (e.g., to mitigate the distress and/or anxiety surrounding or caused by the experience, and to connect with the present moment thus helping the client to enter the optimal arousal zone).

### Basic interventions

#### Opening and direction of the sessions

Not everything regarding psychedelic integration practice is or should be non-ordinary. After all, besides focusing on the psychedelic experience and its consequences, sessions of psychedelic integration are not different from regular counseling, therapeutic meetings or, in specific cases, crisis intervention. Thus, most of the practical considerations here should be taken as usual, based on the practitioner’s workflow. In our understanding, these considerations should include:

- taking care of the client’s *sense of security and confidentiality* (i.e., providing support, non-judgmental space, and being careful to not overwhelm the client with our questions, responses, and/or attitude to begin with);
- co-determining *specific work direction and priorities* (e.g., what brought the client to us? Does the client need/want to analyze and understand the content and outcome of their psychedelic experience or rather to calm down and deal with the adverse symptoms following it?);
- conducting *general medical history and screening*, that might inform about the client’s medical history as well as health condition and socio-economic situation;
- establishing a *formal structure* of the work being undertaken (i.e., length, frequency, number, place, time, and costs or other terms of meetings).

#### One size does not fit for all

We believe that every client must be treated with as much of an individualized approach as we are able to provide them within a given circumstance. In the case of psychedelic integration, this means that we have to reflect on several factors that might inform us about and help us to adjust to a particular client. Based on the results of our roundtable discussions in this regard we propose the key considerations (and exemplary questions that might be useful for assessing them), which are summarized in Table 1.

**Table 1:** Our proposed key considerations and exemplary assessment questions about the psychedelic experience(s) of interest.

Key consideration	Exemplary assessment question
*Setting in which a particular psychedelic experience occurred*	Where did this experience take place? What do you recall from your surroundings? Where were you when under the influence of this substance? Were you alone? Who accompanied you during this experience?
*Substance that induced the experience*	Was it a classical or non-classical psychedelic? What was the dose? Did you redose or take a booster? If so, at what point and what was the dose? Was it mixed with other psychoactive substances?
*Motives and intentions to have that experience*	What was your motivation to take this substance? What were your intentions? Why did you want to have that experience? Was it just out of curiosity, as a means of self-exploration, self-development, as a part of experimental treatment or self-medicalization, or for reasons that might be called hedonistic? If it was intentional, what were the hopes and expectations? Have you done anything specific to prepare for this experience?
*Client’s general health condition*	Do you have any physical or mental health problems? Were your parents or other close relatives ever diagnosed with any medical or mental health problems? Are you or were you taking any medications? Do you have any relational, social, or other problems that concern you?

Further, an individualized approach is needed due to the so-called noetic quality of the psychedelic experience, which refers to a common phenomenon of having a feeling that the content of the psychedelic experience has been as real or even more real than one’s usual sense of reality (Richards, 2015; Yaden et al., 2017). The phenomenon relates to the meaning and level of significance that is attributed to a particular psychedelic experience, which is why it should be taken very seriously. We believe that it might be useful to treat the psychedelic experience as one of the experiences in the client’s life (i.e., not to underestimate its relevance), despite the metaphorical language of its description. However, due to its highly subjective nature, adjusting our approach toward a particular client is necessary in order to provide a successful intervention.

#### Competences and metaskills

Phelps (2017) attempted to develop a list of competencies for the training of psychedelic therapists. According to this list, working with psychedelic experiences may require knowledge of psychedelic effects and mechanisms of action, empathetic abiding presence and trust enhancement, self-awareness, and ethical integrity as well as proficiency in complementary techniques. The author also indicated so-called spiritual intelligence, conceptualized as having not only the awareness of our self and relationships but also of what is beyond (transcendent). As a complement to this, we believe that it should also be emphasized that mental health practice (psychedelic integration included) requires not only learned and crafted competencies but also specific attitudes and ways of working with clients, which range beyond the theoretical knowledge, practical methods, and individual psychotherapeutic approaches. This constitutes not *what* but *how* the practitioner is working (i.e., managing the pre- and post-session contact, using humor or self-disclosure), what has been referred to as *metaskills* (Mindell, 1991). Therefore, we suggest that specialists willing to provide psychedelic integration should act toward the development of both such competence and meta-skills, as they may contribute to more manageable and effective practice.

#### Resource orientation

In our view, psychedelic integration practice should be based on the client’s resources (i.e., source of support, strength, or a coping strategy), rather than their deficits. According to this strength-based approach, it might be useful to encourage the clients to explore their own biography for skills, personal values, experiential knowledge, passion, good memories, favorite activities, and other sources alike. Those are the client’s resources that might serve to mitigate, compensate or combat his/her vulnerability, as well as to cope with the difficulties that do or will occur (i.e., Priebe et al., 2014). In fact, even exploring these resources might lead to weaving a new narrative or meaning and thus contribute to the integration of the psychedelic experience of interest.

It is also important to search for the outer resources, which might include the client’s social network and surroundings, such as family members, friends, or others whom clients might trust, and speak about this difficult situation/experience without feeling ashamed or judged. Such resources may also contribute to a client’s strength, as they might serve as a stabilizer in challenging situations or conditions. In fact, if the client has such relational resources, the integration of a psychedelic experience that did not result in adverse symptoms usually does not require any support from mental health specialists.

One particular resource-oriented intervention that might be helpful in this context is to bring the client’s attention to their “inner healing” part (see “Trust inner healer or be directive?” in the Theoretical consideration section), which might be identified by the fact that it’s what encouraged him/her to seek help (though it is not always the case). It might also be referred to as the source of the client’s hope and optimism, or as what helped him/her to overcome some past difficulties. Identification of such a “healing part,” preferably felt rather than just understood, should be then empowered as a source of constant support that might accompany clients’ present and subsequent processes.

#### Calming down and grounding

Although usually needed during or immediately after a psychedelic experience, calming down and grounding techniques might also be useful in the course of psychedelic integration practice. For instance, this might include situations when the client contacts us due to experiencing undesirable symptoms, or when he/she will reconnect with the psychedelic experience during sessions. Rapid speech, restlessness or agitation, and psychotic-like symptoms might indicate a client’s need to calm down and obtain reassurance before undertaking any other interventions. The aim of such techniques is to mitigate the distress and anxiety surrounding or caused by the experience and to connect with the present moment thus helping the client to enter the optimal arousal zone. Hence, these techniques may both help clients carry on with everyday duties and provide the space necessary for further work with the psychedelic experience.

There are many ways in which calming down and grounding after a difficult psychedelic experience might be facilitated. An example is bringing the client’s attention to his/her feet on the floor/ground, looking around and describing the immediate surroundings, performing a body scan, stretching, mindful walking, direct contact with nature (i.e., working with plants in the therapeutic context or barefoot walking on the grass/ground or gardening, when outside). Another example might be the active imagination or focusing methods (i.e., encouraging the client to imagine a safe space or exploring and describing his/her bodily sensations in the present moment). Importantly, this does not mean that any redirection of attention is favorable (e.g., engaging in social media or other screen time is discouraged). Further, self-expression activities, such as writing, singing, painting, or dancing are also highly recommended. In addition, if needed, the client should receive some sort of general self-care and health-promoting recommendations regarding sleep, diet, stress management, screen time moderation, etc.

#### Normalization and psychoeducation

Due to the nature and broad spectrum of psychedelic effects and their possible outcomes, the clients that we approach may have experienced themselves in a way they never had before, might be worried about their mental health, or even that their altered condition or related dysfunction will last for the rest of their life. Therefore, normalization might help to manage their stress by seeing that their concerns are actually common among people who have also experienced it. This should be evidence-informed psychoeducation about psychedelics effects, mechanisms of action, afterglow phenomenon, general safety, related risks, and consequences, as well as how set and setting influence the psychedelic experience (i.e., as provided in the Theoretical consideration section). Ideally, the specialist would be able to refer to the current scientific literature in this field, preferably meta-analysis results. Also, in specific situations, we believe that self-disclosure about the psychedelic experience (e.g., in a research setting) can facilitate normalization and a sense of trust, and thus be helpful for the client. However, care must be taken in doing so to avoid overlooking or devaluing the distress experienced or needs expressed by the client. Here, the occurrence of HPPD or other psychiatric conditions (e.g., psychotic symptoms) should not be underestimated, and we advise referring the client for a psychiatric consultation if such symptoms are present, while still providing support and addressing other existing difficulties.

#### Analyzing the experience

When the abovementioned concerns are checked and taken care of, it is time to address the very content of the client’s psychedelic experience. This is not about our interpretation but about the facilitation of the client’s process of understanding and drawing conclusions about a particular experience and its consequences. Such facilitation should include providing a safe, non-judgmental space to describe the experience in detail, paraphrasing and otherwise supporting the sharing of the content, perceptions, and feelings of a particular experience. This might be followed by asking open-ended, in-depth questions, or inquiries, in order to explore the outcomes or impact that the psychedelic experience may have already had on the client’s life, as well as the insights and potential changes that may yet occur. We recommend expressing curiosity during such a process. Further, oftentimes it might also be useful for a client to provide him/her a space and the instruction to reflect on a psychedelic experience of interest. This might include reconnecting with the experience and drawing conclusions from it after coming back to the present moment. Table 2 provides our proposition of the exemplary instructions and questions that may support fulfilling each of those steps. This particular set of questions resulted from our roundtable discussion and is based on currently used protocols of clinical trials with psychedelics in the National Institute of Mental Health, Czech Republic.

**Table 2:** Our proposed exemplary instructions and questions are useful in the facilitation of reconnection with, going back, and drawing conclusions from the client’s psychedelic experience.

Focus on the past – reconnecting with the experience.
Instruction: *Ask the client to briefly close his/her eyes, take a few deep breaths and then try to recall the most important moments of his/her psychedelic experience, then ask the following questions.*
What’s the first thing that comes to your mind when focusing on that experience? What did you perceive as the most pleasant thing during the experience? Have you had any similar experiences before? (not necessarily related to psychedelics) What helped you cope with difficult moments? Which moments of the experience were the most important for you?
Focus on the present – going back from the experience.
Instruction: *Ask the client to stay with his/her closed eyes, focus attention inwards before answering the following questions regarding the previously reconnected experience, and open his/her eyes again (encourage the client to provide as many details as possible).*
Can you describe your current impressions? What are you thinking about now? What is now going on in your body? What are your feelings/emotions? How do you feel about reality?
Focus on the future – drawing conclusions from the experience.
Instruction: *Ask the client to briefly close his/her eyes for another moment and think about the experience in the context of his/her life in the near future, then ask the following questions.*
How would you rate the realness of your experience? Was it an authentic experience, or just the effects of the substance, which is now gone? Is there something you can bring back to your everyday life from this experience? What needs to be done in order for it to happen? What would need to happen for you to feel that this experience got resolved or integrated?

In addition to the experience itself, the client’s prior intentions and/or expectations for it can also be a valuable subject of analysis, with particular attention to how they relate to the actual content and outcome of the client’s experience. Importantly, such psychedelic experience analysis does not need to be limited to the above-mentioned “talk-therapy” interventions. We advise supporting it in a more expressive manner, such as by drawing, writing (both during the session or as “homework”), role-playing, dancing, or other forms of spontaneous movement (depending on what the client and we ourselves are comfortable with).

#### Organizing the experience

Further, as the psychedelic experience may be remembered as a series of incidents rather than a coherent process, the client might need and benefit from an effort to organize it. Organizing the experience might support additional insights by enlarging the context and perspective. Thus, we believe that it might be helpful to provide the client with certain “maps.” Following are a few examples of such exercises that are based on our clinical practice experiences.

The simplest example of a map to organize the psychedelic experience would be the axis of time. For this purpose, we can provide the client with a piece of paper and a pen, ask him/her to draw a timeline, and mark on it the parts of the experience that he/she remembers. Then we might ask him/her to put additional points on a timeline, such as making a decision on substance intake, coming to us, and whatever relevant events he/she might think of in between.

**Figure 4:**
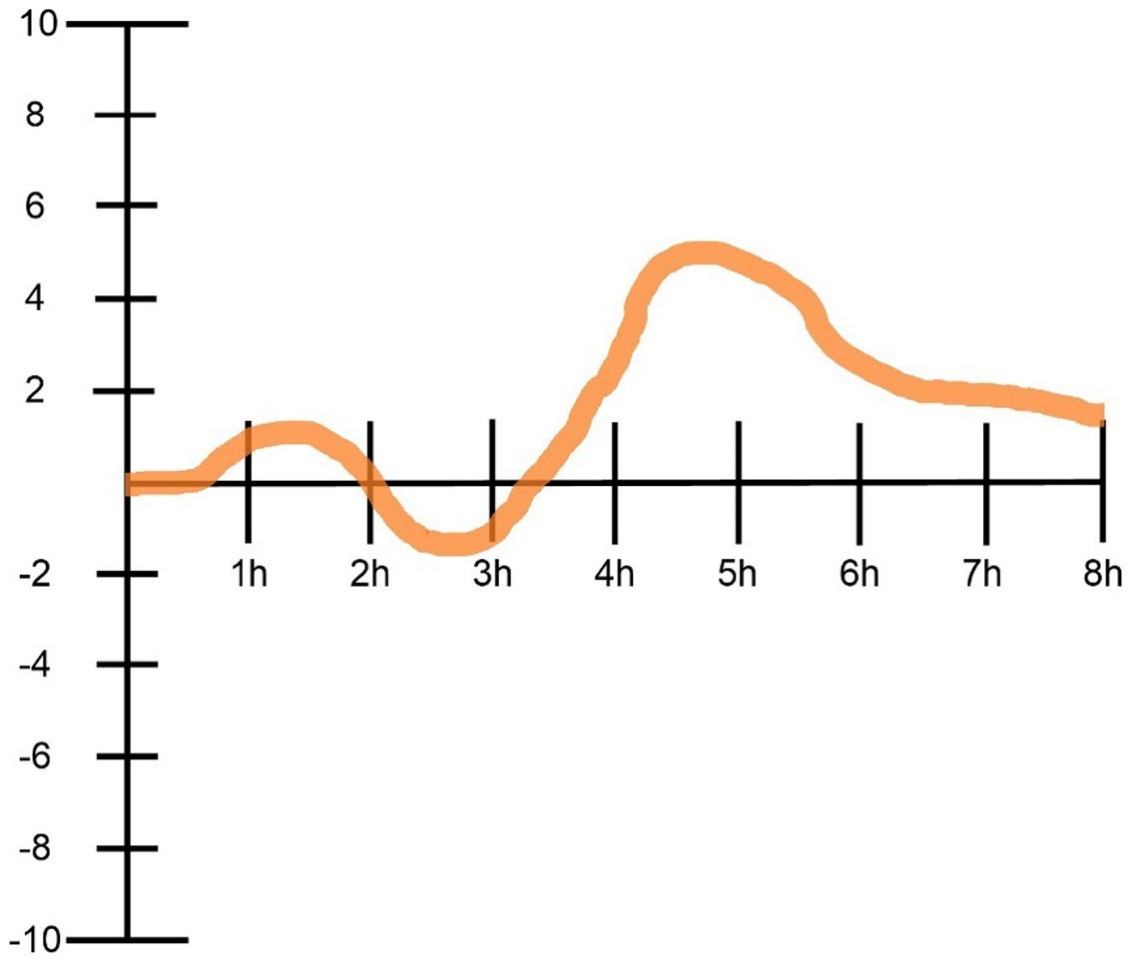
An example implementation of the “graph of emotions” exercise for organizing psychedelic experiences according to emotional intensity as it fluctuated over time. In this case of the generally positive psychedelic experience, we see that after about an hour of substance intake (psilocybin), the person felt elevated (+1), which however quickly became uncomfortable (-2) for about an hour, after which it began to gradually but quite dynamically elevate, peaked between the fourth and fifth hour (+5), and then was progressively “going back to normal” for the next few hours.

Likewise, a client might be asked to draw and fill in a “graph of emotions,” where the X axis represents the given psychedelic experience timeline (e.g., from the psychedelic intake or some time before to the time at which the effects of a particular psychedelic substance usually subsides), and the Y axis represents a scale of emotions intensity (e.g., from. +10 to −10). Clients can also be encouraged to make notes about important moments or emotions. Such a “graph” can also be used to organize and explore the intensity of perceptual changes or other elements of the psychedelic experience the client refers to as salient. Figure 4 depicts an example of such exercise implementation.

Another useful way of organizing the experience might be by a reference to the body (i.e., in terms of bodily sensations accompanying the client during the experience, or any repetitive sensations associated with the experience). The client’s relationships are also worth exploring for any influence on the psychedelic experience (i.e., how are they sustained or if there are any ongoing revisions or movements). Furthermore, if there were other people involved in the psychedelic experience, is it reasonable to consider how the client sees them or the relationship with them now.

### Specific interventions

#### Progression to the second stage

The assumption behind our two-stage model of psychedelic integration is that for some clients, perhaps the majority, the abovementioned interventions (constituting the first stage) should be enough to meet their needs and goals. However, others may need extended support and more sessions, which will usually require meetings applying more specific methods of psychotherapeutic support. This need might occur primarily when the client is experiencing adverse symptoms associated with challenging psychedelic experiences that have not yet been resolved, or when previous work triggered content (e.g., autobiographical or relational) that causes distress and thus requires addressing. Interventions that can serve such purposes range from approaches developed specifically to work with non-ordinary states of consciousness (i.e., indigenous practices or Holotropic Breathwork), through models of integration of psychedelic experiences, to utilization of the general psychotherapeutic approaches. These are briefly described and discussed below.

#### Models and approaches to psychedelic integration

Although psychedelic integration was acknowledged and practiced in some form since the mid-1950s (for review see: Aixalà, 2022), well-elaborated models of integrating psychedelic experiences are being developed only very recently, with publications in this regard going back no further than 2017 (Bathje et al., 2022). These include a quite diverse spectrum of models that are either derived from or refer to specific psychotherapeutic approaches. This is understandable as a client’s needs and goals regarding psychedelic integration do not usually differ from those with which clients generally seek help from mental health specialists. The exceptions apply to experience dynamics and its content that might be described as psychedelic-specific. However, while psychedelic experiences substantially exceed everyday cognition and emotions, they still fall within the wide range of human experience. Hence, it is reasonable to apply various psychotherapeutic approaches to work with the content and consequences of psychedelic experiences, which have already been recognized by a number of authors.

As depicted in Table 3, most of the currently available models of psychedelic integration can be described as holistic, as they combine methods oriented toward the psychological, somatic, spiritual, and community/environmental domains, while other models concentrate solely on mind and behavioral change. The most commonly used therapeutic approaches are transpersonal psychology, Jungian analytical psychology, and third-wave cognitive-behavioral approaches (i.e., Acceptance and Commitment Therapy). Some of these models are also based on indigenous traditions, in which psychedelic substances were commonly utilized for a variety of purposes. Here it should be stressed, however, that practices derived from indigenous traditions might be difficult or even potentially inadequate for implementation outside the specific setting, and rituals that these practices emerge (Yaden et al., 2022b).

**Table 3:** An overview of current models of psychedelic integration, with an emphasis on their overall purpose, the therapeutic approaches they are based on, as well as the specific domains of human experience they focus on, and the stages or major themes of integration work they distinguish.

Model, (author, year)	General purpose	Approaches	Domains	Stages/themes
Psycho-Spiritual Integration Process (Cohen, 2017)	Establishment or reunification of the harmonic relationship between the conscious and unconscious	Jungian analytical psychology, Ayahuasca ceremonies tradition	Psychological, somatic, psychospiritual	Pre-ceremony life experiences, the ceremony, post-ceremony integration, process and practices during and after integration, and ayahuasca itself
Visionary Plant Medicine Integration (Coden, 2017)	Providing a manual for people participating in ceremonies with plant-based psychedelics	Transpersonal psychology, Indigenous shamanic traditions	Reflection, Inner Listening and Creative Expression, Psychospiritual Practice, Meaning Making, Spaciousness and Time, Nature and Grounding, Physical Care, and Cultivating Virtues and Turning Outward	Introspection, self-care, relationships and community, the natural world
Holistic Model for a Balanced Life (Bourzat and Hunter, 2019)	Moving oneself and one’s life in the holistic direction	Indigenous shamanic traditions	Body, Mind, Spirit, Community, and Natural Environment	Returning from the psychedelic experience (capturing a narrative on it), understanding the experience, and implementing its decoded content
Realms of Integration (Buller and Moore, 2019)	Providing a self-help manual (workbook) for psychedelic integration	–	Mental/intellect, Mind–body surroundings, Spiritual, Lifestyle/career, Relationships	–
SAFETY (Westrum and Dufrechou, 2019)	Providing general introduction and a self-help manual for psychedelic integration	Based on but not limited to Transpersonal psychology	Psychological, Somatic, Spiritual/existential, Social/Communal	–
Nature Contact (Gandy et al., 2020)	Enhancement of nature relatedness	Nature-focused rituals, nature-based settings, mindfulness practices	Psychological, Affective, Mystical/Awe, Nature relatedness	–
Psychedelic Harm Reduction and Integration (Gorman et al., 2021)	Providing a transtheoretical framework and rationale for clinical practice with clients who using or considering using psychedelics in any context	Transtheoretical: Harm reduction approach, psychedelic-assisted therapy, mindfulness-based and psychodynamic psychotherapy	Psychological, somatic, spiritual/mystical, harm reduction (as a goal of behavioral change)	Preparation (assessment, psychoeducation, harm reduction) and integration (working with challenging and/or positive experiences)
Accept, Connect, Embody (Watts and Luoma, 2020)	Providing a clinical framework for psychedelic-assisted therapy (including preparation and integration)	Acceptance and Commitment Therapy	Psychological, Behavior Change	Pulling together the narrative (giving space to share the experience and support meaning-making), distilling key insights, and supporting behavior change
Beyond the narrow life (Ortigo, 2021)	Providing a philosophical and psychotherapeutic framework for psychedelic-assisted therapy (including preparation and integration)	Jungian analytical psychology, Transpersonal psychology, Existential philosophy, et al.	Psychological, Somatic, Spiritual, Behavioral	Beyond personal identity (integrated, flexible self), beyond shadow projection (compassion), beyond the narrow life (creative engagement and symbolic renewal)
Psychotherapy for non-ordinary states of consciousness (Aixalà, 2022)	Providing general-purpose guidelines for psychedelic integration	Transpersonal psychology, Holotropic Breathwork, Gestalt therapy, et al.	Cognitive, Emotional, Behavioral, Somatic, Spiritual, Social, Time	–
EMBARK (Brennan and Belser, 2022)	Providing a transdiagnostic model of psychedelic-assisted psychotherapy	Cognitive-Behavioral Therapies, Mindfulness, Motivational Interviewing, et al.	Existential-spiritual, Mindfulness, Body-aware, Affective-cognitive, Relational, Keeping Momentum	–
Internal Family Systems Model for Psychedelic Experiences (Morgan, 2020)	Providing a framework for understanding the psychedelic experience and helping embody its insights into everyday life	Internal Family Systems	Psychological, Emotional, Somatic, Behavioral, Spiritual	Preparation, Experience, Integration
Psychedelic- supportive psychotherapy (Wolfson, 2023)	Provide a model of psychological support that can be immediately implemented by qualified practitioners	Transtheoretical with the emphasis on harm reduction approach and positioning the therapeutic alliance as a central agent of change	Emotional, Psychological, Spiritual	Before and beyond the psychedelic experience

A detailed presentation of current psychedelic integration models is beyond the scope of this paper. Here we focused only on a brief description and discussion of how selected psychotherapeutic approaches might be utilized for psychedelic integration. Whereas in Table 3 we summarized all of the currently available models of psychedelic integration by specifying their general purpose, utilized therapeutic approaches, domains of human experience that they focus on, stages or major themes of psychedelic integration that they distinguish as well as model label, author(s) and year of publication. This will hopefully inform readers about the scope of models that might potentially be implemented within the individual’s practice. The summary provided is based on our literature review, as well as a recently published review in this regard (Bathje et al., 2022), with the inclusion of Morgan (2020), which we found was omitted, as well as work by Aixalà (2022) Brennan and Belser (2022) and Wolfson (2023), that were published after the compilation of the Bathje et al. (2022) review (in August 10, 2021).

Importantly, the authors of individual models presented in Table 3 provide also examples of some specific practices serving to facilitate psychedelic integration. These practices form a wide and diverse range of methods used in the given psychotherapeutic approaches, some of which were selected by use and briefly described below. It should be stressed, however, that the provided list of examples is by no means exhaustive, and that professional training (usually certification) as well as supervision may be required to apply them when working with clients. In addition, the current models of psychedelic integration also include a range of more general practices that are often used outside the clinical or psychotherapeutic setting, including self-care. Among these practices are: creative expression, journaling, reflection, musical engagement, spending time in nature, active movement, relaxation, meditation, breathwork, and spiritual practices (for review see: Bathje et al., 2022).

##### Acceptance and commitment therapy

More than one manualized protocol for combining ACT with psychedelic-assisted psychotherapy for the purpose of depression treatment was proposed, including the psychedelic integration practice (Guss et al., 2020; Sloshower et al., 2020; Watts and Luoma, 2020). This was based on the theorized overlap between the ACT’s main objective of reinforcing psychological flexibility and the potential therapeutic mechanisms of psychedelic-induced experiences (Davis et al., 2020). Although several differences exist, in these protocols the therapist is first focused on eliciting the client’s narrative of the psychedelic experience and then attempts to identify parallels between this narrative and the principles of ACT (i.e., present moment, contact, acceptance, self as a context, diffusion and committed action). The ACT-specific integration practices that the therapist undertakes to facilitate psychedelic integration include practicing mindfulness, reflecting on changes that have occurred due to the experience, discussing the client’s values and their current implementation in his/her life, reinforcing internalization of desired changes, and forming of new habits. For more information on the utilization of ACT and other third-wave CBT approaches to psychedelic integration, we refer to the review by Yaden et al. (2022b).

##### Mindfulness-based interventions

In addition to above mention basic calming down and grounding techniques, practicing mindfulness-based interventions may facilitate both minimizing adverse symptoms (e.g., by alleviating/managing stress or anxiety) and maximizing benefits (e.g., as a tool for bringing greater awareness, balance, or self-control to everyday life) resulting from client’s psychedelic experience(s). Mindfulness might be defined as both the simple relaxation technique and the psychological process of bringing one’s attention to whatever experiences occurring in the present moment, exploring senses, thoughts, and emotions with a curious and non-judgmental attitude (e.g., Brown and Ryan, 2003). Examples of mindfulness practices include mindful breathing, walking or eating, and body scan meditation. Though “mindfulness” derives from contemplation and philosophical traditions, it was developed in a way that individuals can effectively practice it in the absence of such traditions or related vocabulary. To date, a growing body of evidence has demonstrated both physical- and mental-health benefits of practicing mindfulness in various patient conditions, with a particular clinical-oriented focus on reducing stress and anxiety (Grossman et al., 2004), as well as supporting well-being (Allen et al., 2021). Whereas recent attempts have been made to combine mindfulness-based interventions and psychedelics in clinical practice (Payne et al., 2021). This includes utilizing mindfulness practices to both prepare for a non-ordinary and potentially challenging state of consciousness resulting from psychedelics, as well as to enhance psychedelic integration (e.g., by deepening, embodying, and maintaining the novel perspectives and motivation instigated by psychedelic experience).

##### Internal family systems

IFS is a psychotherapeutic approach based on the non-pathologizing assumption that the human psyche is composed of inner parts that, despite emerging and operating in a congruent direction (that is, the protection of the inner Self), can come into conflict with each other, which might result in dysfunctions and psychopathologies. Morgan (2020) proposed the utilization of the IFS model in working with psychedelic experiences, both before (i.e., preparation), during, as well as after (i.e., integration) their occurrence. Regarding the latter, the work should focus on the client to achieve harmony between his/her parts, which might be facilitated by recognition and acknowledgment of individual inner parts, and engage in the dialog both with and between them (through specific techniques of asking questions to the client or instructing him/her to redirect attention and ask questions to a given part). A range of supporting practices might also be prescribed, such as self-expression by movement or creative work (e.g., drawing), breathwork, or guided meditation considering specific parts. In general, psychedelic integration practice utilizing this approach concerns IFS-parts that were active around the psychedelic experience. For instance, during the psychedelic experience, the client may have gained access to his/her vulnerable part (i.e., the Exile) that needs care and release from, e.g., a sense of exclusion. In this case, a psychedelic integration would be focused on connecting with and unburdening this part of the client.

##### Process-oriented psychotherapy

According to POP, human experience flows through different senses, or channels, where each sensation is a signal from the unconscious (Mindell, 1985). It might be seen, heard, or felt, as well as be described by movement, relationships with other people, groups, workplace, and institutions (so-called “world channel”). Psychedelic experiences, by definition, involve being exposed to unconscious content, and often lead to client’s higher sensitivity to the processes that might potentially unfold in their daily life and through different channels (Pavlovič, 2020). The aim of process-oriented work is to amplify the signal and recreate it in matters of feeling and experiencing it with awareness, so it would provide meaningful insight. This amplification is facilitated by a specific process-oriented technique of following the manifestations of the unconscious through different channels to extract its full content. But effective and often used techniques here are also role plays. A good example is the empty chair exercise, a technique used also in the Gestalt therapy approach, in which the client becomes a role and interacts with other roles, i.e., characters from a psychedelic experience, dream, or client’s life, represented by one or more empty chairs in the room.

##### Working with traumatic-like symptoms

The intense, confusing, and often overwhelming nature of the psychedelic experience might be very challenging and result in traumatic-like symptoms. By these, we mean symptoms that are similar to those of acute stress disorder (Bryant, 2017). These include hyperarousal, re-experiencing, or dissociation, which often lead to distress or dysfunction (e.g., sleep problems, intrusive thoughts, decreased mood), that are commonly the reason for seeking help in the first place (Aixalà, 2022). Currently, MDMA-assisted psychotherapy is a promising emerging treatment for Post-Traumatic Stress Disorder (Mitchell et al., 2021), but there is no evidence-based intervention to target psychedelics-induced symptoms of trauma. However, due to its similarities with other better known cases of trauma symptomatology (e.g., accident-, assault- or combat-related trauma), it is reasonable to treat a person with trauma originating from a psychedelic experience as we would treat someone who has experienced a different type of traumatic event (e.g., Badour and Feldner, 2013). Thus, we believe that well-established evidence-based interventions, such as cognitive processing (Resick et al., 2016), narrative and prolonged exposure therapy (Mørkved et al., 2014), or Eye Movement Desensitization and Reprocessing (EMDR; Shapiro, 2017), may have a potentially beneficial application. It is also fair to say that, to a certain extent, most psychotherapeutic approaches provide trauma-informed care. Here it should be stressed however, that the use of these approaches and methods requires specific training, as well as that their effectiveness in working with the content or consequences of psychedelic experiences is currently unknown.

##### Psychodynamic approach

The developmental, psychodynamic perspectives on human personality structure might be particularly useful in understanding and dealing with the client’s psychedelic experiences. In fact, it has been hypothesized that the individual dynamics of psychedelic experiences result from the temporary cessation of intrapsychic defense mechanisms (Carhart-Harris et al., 2014; Razvi and Elfrink, 2020), and the psychodynamic perspective itself was used to develop the first conceptualization of psychedelic experiences (Grof, 1976). In general, this perspective views levels of psychic functioning on a psychotic-borderline-neurotic continuum, which can be looked at in terms of personality growth (from vulnerable states into more advanced forms of personality functioning), with level-specific defense mechanisms (McWilliams, 2011). Likewise, the client’s main source of preoccupation (safety > autonomy > identity), quality of experienced anxiety (annihilation > separation > fear of losing control), intrapsychic conflict (i.e., separation/individuation) or ego-state (overwhelmed > fighting > participating in therapeutic alliance), may guide our thinking regarding client’s personality organization and current mental health condition. In cases where premorbid personality functioning points to a rather lower level of organization and observing ego quality is missing (which in this context can be called psychotic), it may be necessary to pay greater respect for the client’s defense mechanisms, as their deconstruction through inadequate interventions or attempts to return to psychedelic experiences could be destabilizing (which these mechanisms guard the psyche against). Similarly, borderline clients dealing with their psychedelic experiences may find themselves more prone to abandonment and constantly negotiating the therapist’s trust. Therefore, in such cases establishing a safe therapeutic alliance may take several sessions before integration can focus on the psychedelic experience itself. On the other hand, neurotic clients are more likely to establish a therapeutic alliance and concentrate on the meaning of the psychedelic experience in their life, as they typically display a coherent sense of identity and have a better understanding of their internal states and affects. However, it should be noted that understanding a client’s level of personality organization may take time and cannot be assumed based on just one session.

##### Treating psychedelic experiences as dreams

Given that a psychedelic experience can evoke unconscious and symbolic representations of the psyche, we think it is reasonable to approach these experiences as we would treat a significant dream with which a client has come to us. A famous quote from Holden (1980) states that “If, as Freud said, dreams are the royal road to the unconscious, is it possible that psychedelic drugs are a superhighway to the unconscious.” Thus, psychedelic integration could be held in a similar way in which the unconscious material becomes conscious when dreams are interpreted through Jungian analytic or depth psychology (Vedfelt, 2002). Accordingly, one should consider that unconscious material of dreams, and psychedelic experiences alike, might contain not only insight into individual aspects but also into collective data and archetypes that reach beyond individuality and might be common to every human being. In practice, this perspective focuses on interpretations through decoding and amplifying various symbolic and metaphorical events or sensations into conscious, everyday language, thus extracting its potential insights for the client’s life. Notably, the analysis may not need to be limited to the psychedelic experience itself but also include the client’s recent dreams or other events that may hold symbolic value. The aim of such exploration is to build up a story (narrative) of the experience so that it would be manageable, sensible, and meaningful. In addition, the clients may also be invited to create their own fairy tales or personal myth based on that work. This could be told, written, or drawn, as long as it would serve the clients in their future life.

##### Sharing circles

To this moment we were focusing on individual, one-to-one processes of psychedelic integration. However, much of what was already said is applicable to the form of group meetings and sharing circles as well. Such forms of integration were practiced before, in the 1960s and 70s, and are gaining popularity today due to the growing interest in using psychedelics (Fadiman, 2011; Trope et al., 2019). In many organized settings (i.e., psychedelic ceremonies, retreats, or Holotropic Breathwork workshops), sharing circles are formed right after a psychedelic session and/or on the following day, when they are usually combined with participants sharing their intentions before the psychedelic experience, which serves also to established supporting atmosphere between them (i.e., Grof, 2014). Such an atmosphere is common in many forms of group therapy as well, and it is known to be one of the main therapeutic mechanisms of such interventions (Cox et al., 2008). However, integration-focused sharing circles can also be organized in the form of recurring meetings focused on sharing and analyzing past psychedelic experiences. In addition to verbal expression, drawing (e.g., mandala) is often recommended during such circles, which is intended to metaphorically and symbolically reflect the experience and thus serve its integration (i.e., Grof, 2014). Reflections on the experience might be helpful as the everyday language is usually not suitable or enough to provide a satisfying description of one’s experience to others. The opportunity to name and understand the lived experience, receive support and feedback from different perspectives, as well as to engage in spontaneous interpersonal interactions in this regard, can be invaluable in managing it so as to minimize its harm and maximize its potential benefits.

### After the integration

It is often difficult to draw a clear line between psychedelic integration and general psychotherapeutic practice. This difficulty is mostly due to the fact that psychedelics tend to reveal or confront clients with content concerning their biography, patterns of behavior, fears, choices, and life situations, which at the end of the day are not specific only to psychedelics. Nonetheless, we believe that it might be in the client’s best interest to be aware of and reflect on the shifting content of sessions, as it most probably occurs at some point. And, we propose that this shift happens when the session’s content no longer directly concerns the scope of the very psychedelic experience(s), but either deeper or more general client issues. This is not to say that our contact with the client should be discontinued at such a point, as the psychedelic content will probably come back and forth if the meeting becomes long-term. Rather, the client should be informed about our impression of changing the nature of sessions so that he/she might be involved in the decision to continue work (perhaps with different goals or on different terms). This information will also help to avoid confusing the client with psychedelic integration or, for instance, “typical” psychotherapy in which he/she participates.

When it comes to ending such collaboration, however, be it after a successful outcome or for another reason, two general manners should be applied, regardless of the psychotherapeutic approach that was used. First, sum up the benefits and challenges that happened along the way so far, and look upon their constructive effects. This summary serves as, nomen omen, integration of the work that was done. Second, make sure that the client knows what following actions he/she should take in order to sustain well-being, as well as that he/she has contact with a person or place to seek additional support or other help that might be needed in the future due to his/her particular condition.

In a less formal manner, as it is often useful for integrating such processes by giving them an overall title or coining a metaphor, the same might be done in order to close the process of psychedelic integration. To sum up, with our own metaphor, no specialist will integrate someone’s psychedelic experience, but a supportive and informed one might help the client to harvest the fruit that has grown on the trees of their psychedelic experience so that they can take them home, to their daily lives.

## Limitations and recommendations for future guidelines development

We believe that our approach to attempting the development of psychedelic integration guidelines for mental health specialists has proved effective and might inform future attempts to either further expand on these or develop new guidelines in this regard. Therefore, here we identify and discuss both strengths and limitations of the approach we adopted.

To begin with, among strengths, we can note the use of a relatively complex and collaborative approach to guideline development. Specifically, our approach included developing the portions of the guidelines in the subgroups drawn based on experience in particular areas, reaching a consensus of the full team on the content of the guidelines, and collating thus working out the initial draft of the guidelines with the current state of knowledge on psychedelic integration based on the literature review, as well as undergoing a peer-review process of scholarly journal publication. However, future developments of such guidelines might benefit from adopting other procedures that proved to be effective in decision-making or guidelines development (i.e., expert panels, Delphi technique, or multi-criteria decision analysis).

Further, we believe that the collaboration of a diverse team of practitioners and researchers working with clients having psychedelic experiences in different settings was a strength of our approach. However, the backgrounds, expertise, and work experience of our team certainly influenced the content of the final guidelines (e.g., the range of selected psychotherapeutic approaches and methods). Thus, future attempts to develop guidelines for psychedelic integration practice would benefit from extending the scope of approaches and methods recommended for implementation in psychedelic integration practice. This might include, in particular, somatic-based approaches, systemic approaches, specific contemplative or imaginative approaches, approaches utilizing new technologies (e.g., virtual reality), as well as cognitive-behavioral approaches. Though, examples of the latter were included in our guidelines due to relatively extensive literature in this regard. Moreover, our team could also potentially benefit from including individuals that are experienced in training mental health specialists, as well as individuals who were previously involved in the development of other guidelines for this population.

We also want to acknowledge our impression that psychedelic integration is a relatively young field undergoing dynamic development, which makes it very difficult, if possible, to provide a truly comprehensive list of therapeutic approaches and methods that might be potentially useful in this regard. Importantly, while most of the currently available models and methods of psychedelic integration are based on long-standing practices of indigenous cultures or on relatively well-elaborated and theory-driven psychotherapeutic approaches (Bathje et al., 2022; Yaden et al., 2022b), this moment none of currently existing models (or, in fact, methods) of psychedelic integration is supported empirically. This is due to a lack of studies focused on testing the efficacy of psychedelic integration models/methods. Thus, we encourage future research to be conducted with the aim of examining and evaluating psychedelic integration practice, which would also serve future developments of guidelines in this regard.

## Conclusion

The need for psychedelic integration is compounded by the growing interest in and prevalence of psychedelic use in various contexts, as well as the lack of preparation of a cadre of mental health professionals to provide support in this area. The purpose of this paper was to describe our attempt to develop theoretical and practical guidelines on psychedelic integration for mental health specialists who may meet clients in need of such support. This concerns both the need of minimizing adverse consequences and maximizing potential benefits associated with psychedelic experiences. Presented guidelines were developed based on a series of roundtable discussions among an international group of psychotherapists, psychiatrists, and researchers working in the field, as well as on a review of the current scientific literature in this regard. Although psychedelics have been used by humans for millennia, and then became the subject of Western science more than a century ago, the field of psychedelic integration has only recently gained its dynamic momentum. We hope that this paper will play a valuable role in providing a clinician-friendly synthesis of current knowledge considering psychedelic integration, in order to help navigate both the promises and dangers of the “renaissance” and ongoing “mainstreamization” of these mind-manifesting substances.

## Author contributions

JG, ML, CK, and FT participated in initial developments of the guidelines during dedicated round-table discussions. JG conducted the review of current literature. JG and FT incorporated literature review results into the guidelines and prepared the manuscript. All authors contributed to the article and approved the submitted version.

## Footnotes

^1^This particular group of psychoactive substances is often broken down into classical and non-classical psychedelics. The first consists of substances (i.e., psilocybin, mescaline, LSD, DMT) that act as agonists of the serotonin receptors (mainly 5-HT2A). Whereas the non-classical psychedelics have different mechanisms of action, i.e., increasing the production of serotonin, dopamine and norepinephrine (MDMA), or as the NMDA receptor antagonist (ketamine).

^2^The term “psychedelic” was coined by combining two Greek words psyche – mind or soul, and delos – to manifest or reveal.

^3^https://hub.jhu.edu/magazine/2022/fall/new-era-of-psychedelic-treatment/?fbclid=iwar2avlm2bk2vwq-g9uu9f8r3ucbfa2fhs2dzmztushbazbz--sq5vz4ikly (Accessed June 3, 2023).

^4^https://www.fluencetraining.com/certificate-programs/psychedelic-integration-therapy (Accessed June 3, 2023).
